# Welcome to Freshwater Systems, A Domain of TheScientificWorld

**DOI:** 10.1100/tsw.2001.18

**Published:** 2001-04-04

**Authors:** Karl E. Havens, Lorraine Maltby, Karl K. Ramesh Reddy

## Abstract

With an ever-increasing human population size, increasing human impacts on the natural environment, and a growing scarcity of freshwater resources, the study of freshwater systems has become one of the most critical areas of focus for environmental scientists. Never before has there been such a need for an integrated understanding of lakes, wetlands, and flowing waters. This understanding must cross the boundaries of traditional disciplines, reach a wide international audience, and strongly facilitate the linkage between scientific discovery and its real-world application.

*Freshwater Systems* is an innovative publishing venue for accomplishing these goals. Disciplinary boundaries are crossed by the fact that *Freshwater Systems* is a domain in a large, seamless interdisciplinary science publishing network, *TheScientificWorld*. When authors publish in *Freshwater Systems*, news of their work will rapidly be sent to authors and readers in other domains of the network with similar research interests, whether they are physiologists, ecologists, limnologists, biogeochemists, or environmental scientists. The Editorial Board of *Freshwater Systems* (listed below) was established in order to provide expertise across a wide range of subdisciplines related to freshwaters, and to include strong international representation.

In addition to the publication of research articles that address general subjects, we encourage submission of papers with a more regional focus, the prime consideration being that they represent rigorous science. The journal especially encourages the submittal of review articles, and in the early issues will host a series of invited reviews from leading experts covering the breadth of the discipline. Shorter commentary papers also are welcome, particularly when they provide important new insight into unresolved or controversial subjects. Where strong controversy exists, the Editors will periodically invite representative scientists to write "point-counterpoint" commentaries that can be followed by letters from readers.

One of the most important goals of *Freshwater Systems* is to provide a venue for both pure research articles and those addressing major environmental issues. The Editors encourage submittal of articles that demonstrate strong linkages between science and the practical application of scientific findings. We are dedicated to maintaining the highest standards of scientific peer-review, a rapid review and publication process, a highly flexible publishing platform that will allow for many features not possible with standard journals, and rapid communication of information to a global audience. In summary, *Freshwater Systems* is an opportunity for creative ideas, cross-disciplinary integration, and scientific understanding and application. We look forward to the exciting developments in freshwater science that will occur in this new publication.

**Karl E. Havens**, Ph.D., Principal Editor

**Lorraine Maltby**, Ph.D., Associate Editor

**K. Ramesh Reddy**, Ph.D., Associate Editor

January 2001

## Editorial Board for FRESHWATER SYSTEMS A Domain of TheScientificWorld

### PRINCIPAL EDITOR

**Dr. Karl E. Havens**
Watershed Research and Planning, South Florida Water Management District, U.S.

### ASSOCIATE EDITORS

**Dr. Lorraine Maltby**
Department of Animal and Plant Sciences, The University of Sheffield, U.K.

**Prof. K. Ramesh Reddy**
Soil and Water Science Department, University of Florida, U.S.

### EDITORIAL BOARD MEMBERS

### LAKES

**Dr. Michael S. Allen**
Department of Fisheries and Aquatic Sciences, The University of Florida, U.S.

**Dr. Paul C. M. Boers**
Institute for Inland Water, The Netherlands

**Prof. Richard W. Douglas**
School of Environmental Sciences, University of Ulster at Coleraine, Northern Ireland, U.K.

**Prof. Takehiko Fukushima**
Department of Civil and Environmental Engineering, Hiroshima University, Japan

**Prof. Ursula Gaedke**
Institut für Biochemie und Biologie, University of Potsdam, Germany

**Prof. Asit Mazumder**
University of Victoria, Department of Biology, Canada,

**Dr. Marianne Moore**
Department of Biological Sciences, Wellesley College, U.S.

**Prof. Bernadette Pinel-Alloul**
Departement de sciences biologiques, Universite de Montreal, Canada

**Prof. Val H. Smith**
Department of Systematics and Ecology, University of Kansas, U.S.

**Dr. Ellen van Donk**
Netherlands Institute of Ecology, Center for Limnology, The Netherlands

**Professor Dr. Ping Xie**
Donghu Experimental Station of Lake Ecosystems, The Chinese Academy of Sciences, P.R. China

### WETLANDS

**Dr. Daniel L. Childers**
Florida International University, U.S.

**Prof. Ron DeLaune**
Wetland Biogeochemistry Institute, Louisiana State University, U.S.

**Dr. Dale E. Gawlik**
South Florida Water Management District, U.S.

**Prof. L. Gordon Goldsborough**
Department of Botany, University of Manitoba, Canada

**Prof. Brij Gopal**
School of Environmental Sciences, Jawaharal Nehru University, India

**Dr. Michael R. Smart**
US Army Corps of Engineers, Lewisville, Texas, U.S.

### RIVERS & STREAMS

**Dr. Joseph Culp**
National Water Research Institute, Environment Canada, Canada

**Dr. Laura Davalos-Lind**
Chapala Ecology Station, Baylor University, U.S.

**Prof. David Dudgeon**
University of Hong Kong, China

**Dr. Manuel A.S. Graça**
Departamento de Zoologia, Universidade de Coimbra, Portugal

**Prof. Anne E. Hershey**
Biology Department, University of North Carolina, U.S.

**Dr. Gea-Jae Joo**
Department of Biology, Pusan National University, South Korea

**Prof. Rex L. Lowe**
Department of Biological Sciences, Bowling Green State University, U.S.
